# Supplementary material for: Early Hippocampal Sharp-Wave Ripple Deficits Predict Later Learning and Memory Impairments in an Alzheimer’s Disease Mouse Model
Source: Cell Rep. Author manuscript; Available in PMC 2020 Aug 19. (PMC7437815; doi:10.1016/j.celrep.2019.10.056)
Supplement: Emily Jones_Cell Reports_Supplementary Information [file NIHMS1617716-supplement-Emily_Jones_Cell_Reports_Supplementary_Information.pdf]

Cell Reports, Volume *29*

Supplemental Information

Early Hippocampal Sharp-Wave Ripple Deficits Predict Later Learning and Memory Impairments in an Alzheimer's Disease Mouse Model

Emily A. Jones, Anna K. Gillespie, Seo Yeon Yoon, Loren M. Frank, and Yadong Huang

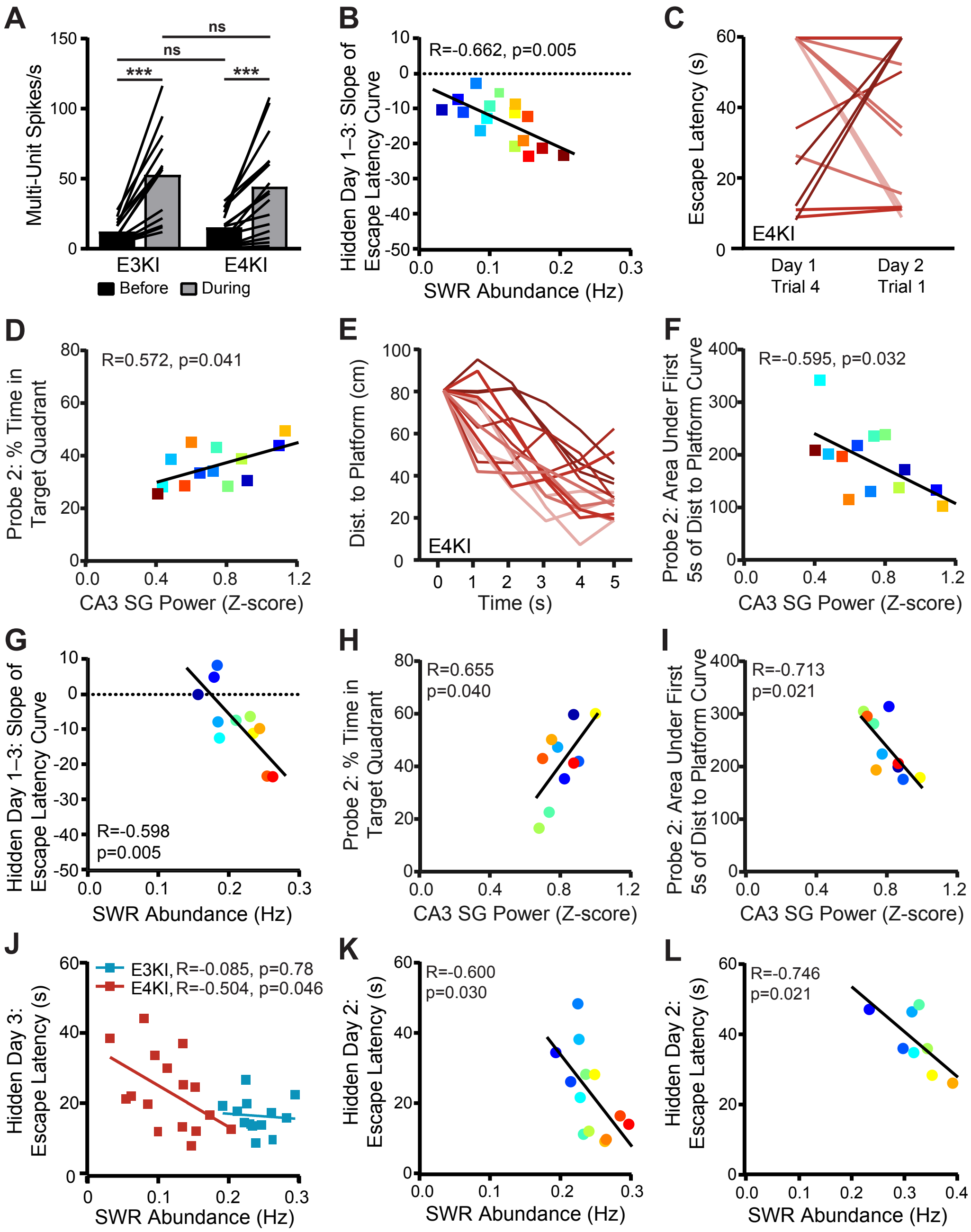
A
Multi-Unit Spikes/s
ns
ns
***
***
E3KI
E4KI
Before
During
B
R=-0.662, p=0.005
Hidden Day 1–3: Slope of Escape Latency Curve
SWR Abundance (Hz)
C
Escape Latency (s)
E4KI
Day 1 Trial 4
Day 2 Trial 1
D
R=0.572, p=0.041
Probe 2: % Time in Target Quadrant
CA3 SG Power (Z-score)
E
Dist. to Platform (cm)
E4KI
Time (s)
F
R=-0.595, p=0.032
Probe 2: Area Under First 5s of Dist to Platform Curve
CA3 SG Power (Z-score)
G
Hidden Day 1–3: Slope of Escape Latency Curve
R=-0.598
p=0.005
SWR Abundance (Hz)
H
R=0.655
p=0.040
Probe 2: % Time in Target Quadrant
CA3 SG Power (Z-score)
I
R=-0.713
p=0.021
Probe 2: Area Under First 5s of Dist to Platform Curve
CA3 SG Power (Z-score)
J
E3KI, R=-0.085, p=0.78
E4KI, R=-0.504, p=0.046
Hidden Day 3: Escape Latency (s)
SWR Abundance (Hz)
K
R=-0.600
p=0.030
Hidden Day 2: Escape Latency (s)
SWR Abundance (Hz)
L
R=-0.746
p=0.021
Hidden Day 2: Escape Latency (s)
SWR Abundance (Hz)

Figure S1. Further predictive relationships identified in the screen cohort and examples of behavioral metrics used in correlations. Related to Figure 2.

(A) Multi-unit activity increases from baseline (400 ms before SWR detection) to during SWRs (0–100 ms after detection) in apoE3-KI ($t(12) = 5.54$, $p = 0.0001$) and apoE4-KI ($t(15) = 4.56$, $p = 0.0004$) mice. No difference between genotypes before ($t(27) = 0.78$, $p = 0.21$) or during ($t(27) = 0.67$, $p = 0.94$) SWRs. N = 13 apoE3-KI and n = 16 apoE4-KI mice aged 12–18 months, paired t test.
(B) SWR abundance predicts slope of escape latency over hidden days 1–3 ($F(1,14) = 10.91$), n = 16 mice.
(C) Individual apoE4-KI mouse escape latency curves over hidden sessions, showing difference between day 1, trial 4 and day 2, trial 1 (overnight), colored from most negative (light) to most positive (dark), n = 16 mice.
(D) CA3 SG power during SWRs predicts percent time spent in quadrant that previously contained the platform on probe 2 ($F(1,11) = 5.34$), n = 13 mice.
(E) Individual apoE4-KI mouse distance to platform curves during the first 5 seconds of probe 1, colored from best (light) to worst (dark) cumulative distance to platform over the curve, n = 16 mice.
(F) CA3 SG power during SWRs predicts area under the curve of the distance to the prior platform location during the first 5 seconds of probe 2 ($F(1,11) = 6.01$), n = 13 mice.
In B–F, apoE4-KI mice aged 12–18 months at electrophysiological recording and 13–19 months at MWM.
(G) In a replication experiment in a separate cohort of animals, SWR abundance predicts slope of escape latency over hidden days 1–3 ($F(1,9) = 13.39$, adjusted $p = 0.021$), n = 11 mice.
(H,I) In a replication experiment in a separate cohort of animals, CA3 SG power during SWRs predicts (H) percent time spent in quadrant that previously contained the platform ($F(1,8) = 6.02$, adjusted $p = 0.04$) and (I) area under the curve of the distance to the prior platform location during the first 5 seconds of probe 2 ($F(1,8) = 8.27$, adjusted $p = 0.06$), n = 10 mice.
In G–I, apoE4-KI mice aged 13–17 months at electrophysiological recording and 14–18 months at MWM. Multiplicity adjusted p values with the Holm-Sidak method.
(J) Example of how SWR abundance for apoE3-KI mice does not overlap with that of apoE4-KI mice and does not predict escape latency for apoE3-KI mice, demonstrating a ceiling effect ($F(1,11) = 0.80$ for apoE3-KI, n = 13; $F(1,14) = 4.77$ for apoE4-KI, n = 16). Mice aged 12–18 months at electrophysiological recording and 13–19 months at MWM.
(K) SWR abundance predicts average escape latency on hidden day 2 ($F(1,11) = 6.19$). N = 13 apoE3-KI mice aged 12–18 months at electrophysiological recording and 13–19 months at MWM.
(L) In a replication cohort, SWR abundance predicts average escape latency on hidden day 2 ($F(1,7) = 8.79$). N = 9 apoE3-KI mice aged 13–17 months at electrophysiological recording and 14–18 months at MWM.
Points colored in order of SWR abundance from blue (lowest) to red (highest). Pearson correlations. Results drawn from 2 independent experiments.

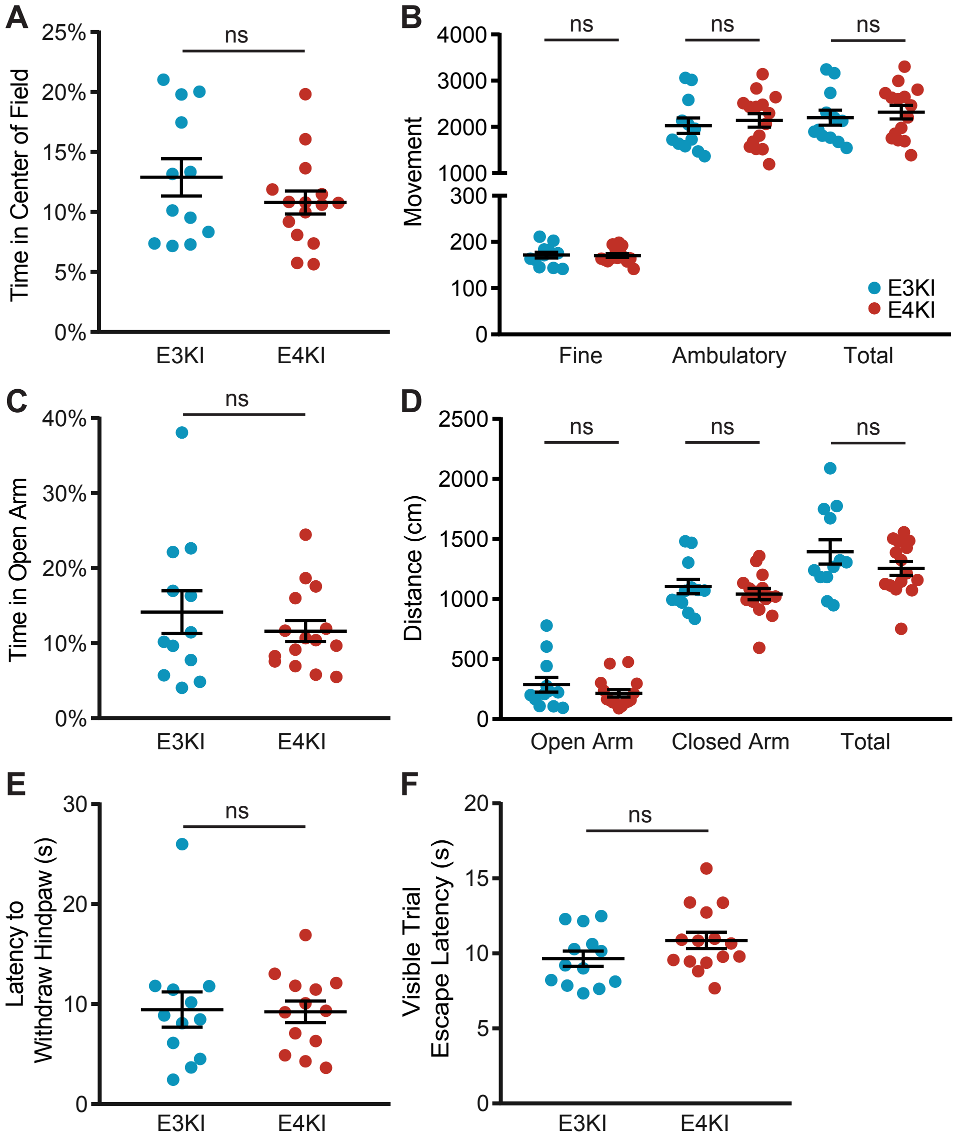
A
Time in Center of Field
25%
20%
15%
10%
5%
0%
ns
E3KI
E4KI
B
Movement
4000
3000
2000
1000
300
200
100
0
ns
ns
ns
E3KI
E4KI
Fine
Ambulatory
Total
C
Time in Open Arm
40%
30%
20%
10%
0%
ns
E3KI
E4KI
D
Distance (cm)
2500
2000
1500
1000
500
0
ns
ns
ns
Open Arm
Closed Arm
Total
E
Latency to
Withdraw Hindpaw (s)
30
20
10
0
ns
E3KI
E4KI
F
Visible Trial
Escape Latency (s)
20
15
10
5
0
ns
E3KI
E4KI

Figure S2. ApoE4-KI mice are not impaired in non-spatial behaviors. Related to Figure 2 and 3.

(A) Percent time in center of open field ($t(25) = 1.19$, $p = 0.24$).
(B) Number of instances of detected movement in the open field ($t(25) = 0.19$, $p = 0.80$ for fine; $t(25) = 0.52$, $p = 0.61$ for ambulatory; and $t(25) = 0.54$, $p = 0.60$ for total).
(C) Percent time in open arm of elevated plus maze ($t(25) = 0.84$, $p = 0.41$).
(D) Distance travelled in elevated plus arms (Mann-Whitney $U = 72.5$, $p = 0.41$ for open arms; $t(25) = 0.81$, $p = 0.43$ for closed arms; $t(25) = 1.25$, $p = 0.22$ for total movement).
(E) Latency to withdraw hind paw on hot plate (Mann-Whitney $U = 66$, $p = 0.54$). N = 12 apoE3-KI and n = 13 apoE4-KI mice, aged 15–20 months.
(F) Escape latency on MWM trials with platform labeled with flag ($t(25) = 1.26$, $p = 0.22$).
N = 12 apoE3-KI and n = 15 apoE4-KI mice, aged 14–18 months, unless otherwise specified. All tests are unpaired t tests unless otherwise specified. Error bars indicate mean ± SEM. Results drawn from 1 independent experiment.

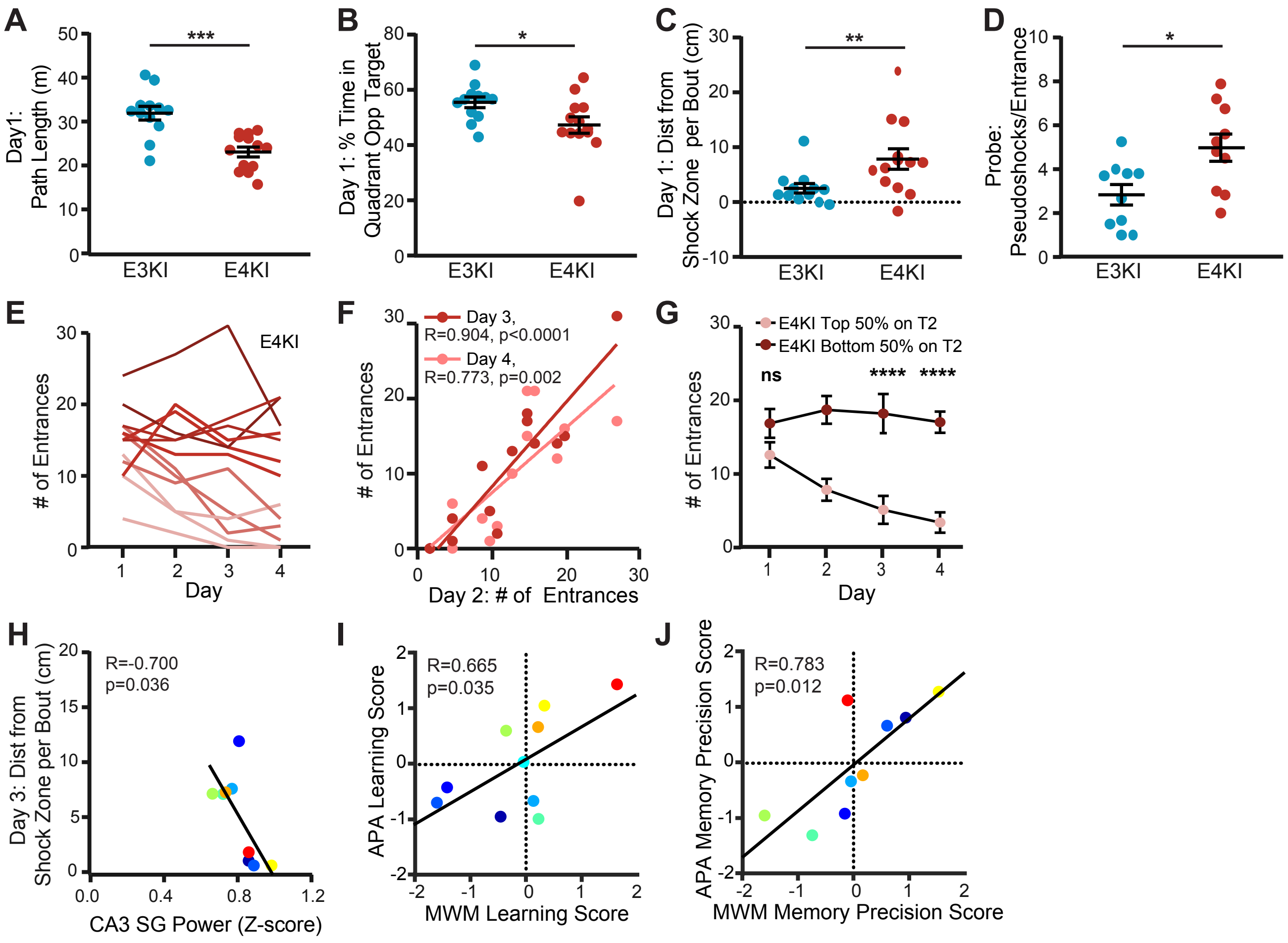
A
Day1:
Path Length (m)
***
E3KI
E4KI
B
Day 1: % Time in
Quadrant Opp Target
*
E3KI
E4KI
C
Day 1: Dist from
Shock Zone per Bout (cm)
**
E3KI
E4KI
D
Probe:
Pseudoshocks/Entrance
*
E3KI
E4KI
E
E4KI
# of Entrances
Day
F
Day 3,
R=0.904, p<0.0001
Day 4,
R=0.773, p=0.002
# of Entrances
Day 2: # of Entrances
G
E4KI Top 50% on T2
E4KI Bottom 50% on T2
ns
****
****
# of Entrances
Day
H
R=-0.700
p=0.036
Day 3: Dist from
Shock Zone per Bout (cm)
CA3 SG Power (Z-score)
I
R=0.665
p=0.035
APA Learning Score
MWM Learning Score
J
R=0.783
p=0.012
APA Memory Precision Score
MWM Memory Precision Score

Figure S3. Aged apoE4-KI mice show impaired acquisition of a spatial avoidance task. Related to Figure 3.

(A) Total path length on day 1 (unpaired t test, $t(23) = 4.64$, $p = 0.0004$).
(B) Percent time spent in quadrant opposite the shock zone on day 1 (unpaired t test, $t(23) = 2.27$, $p = 0.033$).
(C) Distance travelled during each movement bout relative to the shock zone boundary on day 1 (Mann Whitney $U = 30$, $p = 0.008$).
(D) Shocks that would have been received per shock zone entrance during probe were the field electrified; mice which did not enter the shock zone during probe are excluded (unpaired t test, $t(18) = 2.76$, $p = 0.013$).
In A–D, n = 12 apoE3-KI mice and n = 13 apoE4-KI mice, aged 15–20 months.
(E) ApoE4-KI mice show wide variation in performance. Entrances into the shock zone curves colored from best (light) to worst (dark) average performance over all days.
(F) Number of entrances on day 2 predicts number of entrances on days 3 ($F(1,11) = 49.27$) and 4 ($F(1,11) = 16.32$).
(G) Number of entrances into the shock zone for apoE4-KI mice divided into 2 groups based on number of entrances on day 2. Only differences on days 1, 3, and 4 were examined, yielding no difference in day 1 ($t(44) = 1.66$, $p = 0.36$) and significant differences on days 3 ($t(44) = 6.06$, $p < 0.0001$) and 4 ($t(44) = 5.28$, $p < 0.0001$); n = 7 top 50%, n = 6 bottom 50%. Unpaired t tests with Sidak's multiple comparison adjustment.
(H) CA3 SG power during SWRs predicts distance travelled per movement bout relative to shock zone boundaries on day 3 ($F(1,7) = 6.74$); n = 9 mice.
(I) MWM learning performance score predicts APA learning performance score ($F(1,8) = 6.34$); n = 10 mice.
(J) MWM memory precision performance score predicts APA memory precision performance score ($F(1,7) = 11.1$); n = 9 mice.
In E–J, apoE4-KI mice, aged 15–20 months. All correlations are Pearson correlations.
*$p < 0.05$, **$p < 0.01$, ***$p < 0.001$, ****$p < 0.0001$. Error bars indicate mean ± SEM. Results drawn from 1 independent experiment.

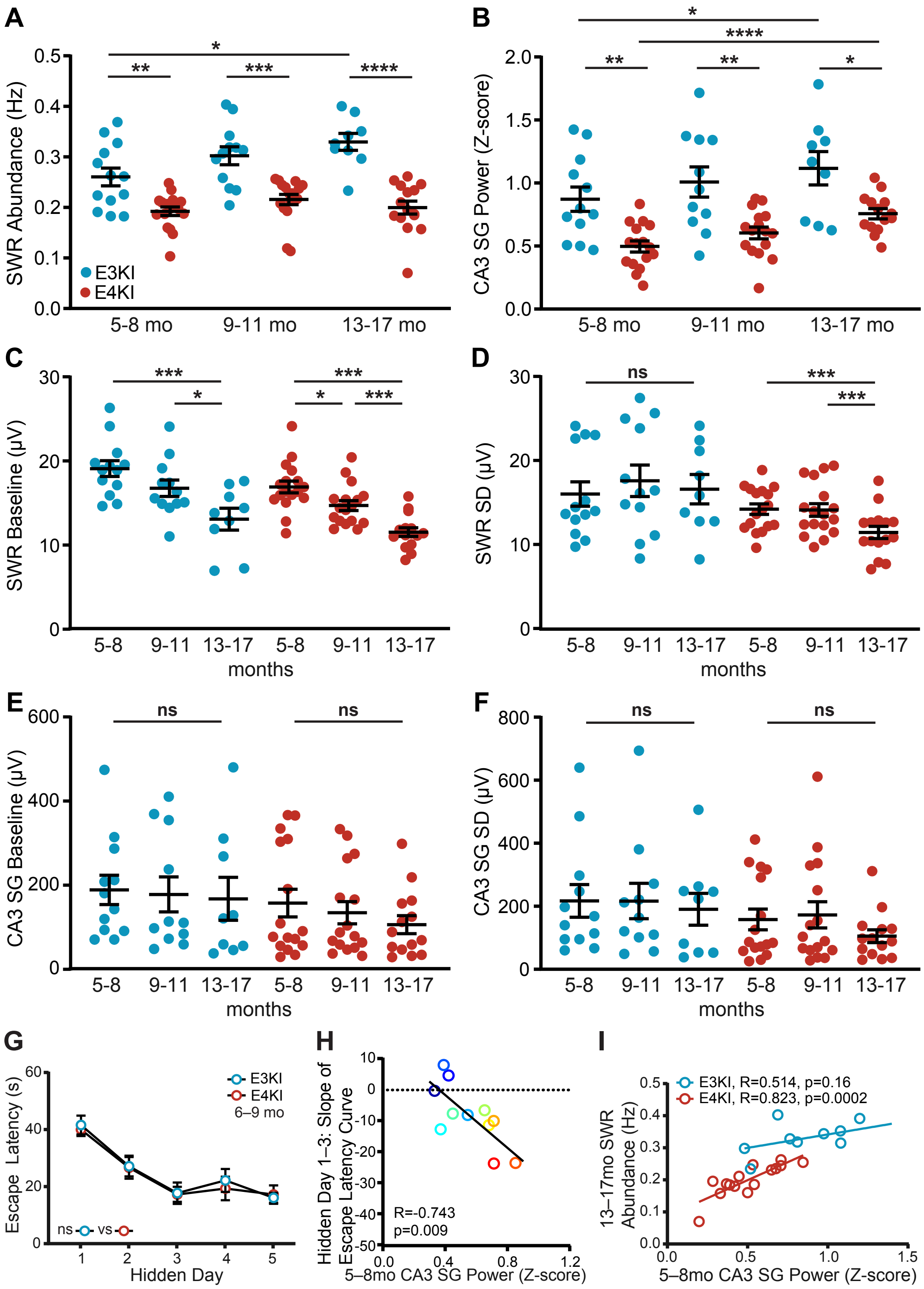
A
SWR Abundance (Hz)
0.0
0.1
0.2
0.3
0.4
0.5
E3KI
E4KI
*
**
***
****
5-8 mo
9-11 mo
13-17 mo
B
CA3 SG Power (Z-score)
0.0
0.5
1.0
1.5
2.0
*
****
**
**
*
5-8 mo
9-11 mo
13-17 mo
C
SWR Baseline (µV)
0
10
20
30
***
*
***
*
***
5-8
9-11
13-17
5-8
9-11
13-17
months
D
SWR SD (µV)
0
10
20
30
ns
***
***
5-8
9-11
13-17
5-8
9-11
13-17
months
E
CA3 SG Baseline (µV)
0
200
400
600
ns
ns
5-8
9-11
13-17
5-8
9-11
13-17
months
F
CA3 SG SD (µV)
0
200
400
600
800
ns
ns
5-8
9-11
13-17
5-8
9-11
13-17
months
G
Escape Latency (s)
0
20
40
60
E3KI
E4KI
6–9 mo
ns
vs
1
2
3
4
5
Hidden Day
H
Hidden Day 1–3: Slope of Escape Latency Curve
10
0
-10
-20
-30
-40
-50
R=-0.743
p=0.009
0.0
0.4
0.8
1.2
5–8mo CA3 SG Power (Z-score)
I
13–17mo SWR Abundance (Hz)
0.0
0.1
0.2
0.3
0.4
0.5
E3KI, R=0.514, p=0.16
E4KI, R=0.823, p=0.0002
0.0
0.5
1.0
1.5
5–8mo CA3 SG Power (Z-score)

Figure S4. Properties of SWRs and associated SG power in CA3 over aging in apoE3-KI and apoE4-KI mice. Related to Figure 4.

(A) SWR abundance of apoE3-KI mice is higher than in apoE4-KI mice across all ages. Two-way mixed-effects analysis of aligned rank transformed data shows significant effect of genotype ($F(1,28) = 43.99$, $p < 0.0001$) and post-hoc Mann-Whitney U test with Sidak adjustment shows significant difference at 5–8 months ($U = 43$, $p = 0.012$, n = 13 apoE3-KI and n = 17 apoE4-KI mice), 9–11 months ($U = 26$, $p = 0.0012$, n = 12 apoE3-KI and n = 17 apoE4-KI mice) and 13–17 months ($U = 5$, $p < 0.0001$, n = 9 apoE3-KI and n = 15 apoE4-KI mice). SWR abundance increases over aging in apoE3-KI mice ($t(19) = 2.926$, $p = 0.028$, n = 13 for 5–8 months, n = 9 for 13–17 months).
(B) CA3 SG power during SWRs in apoE3-KI mice is higher than in apoE4-KI mice across all ages. Two-way mixed-effects analysis of aligned rank transformed data shows significant effect of genotype ($F(1,26) = 12.86$, $p = 0.0014$) and post-hoc Mann-Whitney U test with Sidak adjustment shows significant difference at 5–8 months ($U = 31$, $p = 0.0054$, n = 12 apoE3-KI and n = 16 apoE4-KI mice), 9–11 months ($U = 35$, $p = 0.024$, n = 11 apoE3-KI and n = 16 apoE4-KI mice) and 13–17 months ($U = 31$, $p = 0.0456$, n = 9 apoE3-KI and n = 14 apoE4-KI mice). CA3 SG power during SWRs increases over aging in apoE3-KI mice ($t(18) = 3.14$, $p = 0.017$, n = 12 for 5–8 months, n = 9 for 13–17 months) and apoE4-KI mice ($t(28) = 5.66$, $p < 0.0001$, n = 16 for 5–8 months, n = 14 for 13–17 months).
(C) Baseline across the SWR frequency band decreases over aging in apoE3-KI mice ($t(19) = 4.60$, $p = 0.0006$, n = 13 for 5–8 months, n = 9 for 13–17 months; $t(19) = 2.81$, $p = 0.033$, n = 12 for 9–11 months, n = 9 for 13–17 months) and apoE4-KI mice ($t(30) = 7.25$, $p < 0.0001$, n = 17 for 5–8 months, n = 15 for 13–17 months; $t(30) = 3.10$, $p = 0.012$, n = 17 for 5–8 months, n = 7 for 9–11 months; $t(30) = 4.26$, $p = 0.0006$, n = 17 for 9–11 months, n = 15 for 13–17 months).
(D) SD across the SWR frequency band decreases over aging in apoE4-KI mice ($t(30) = 4.34$, $p = 0.0004$, n = 17 for 5–8 months, n = 15 for 13–17 months; $t(30) = 4.18$, $p = 0.0007$, n = 17 for 9–11 months, n = 15 for 13–17 months), but not apoE3-KI mice ($t(19) = 0.25$, $p = 0.99$, n = 13 for 5–8 months, n = 9 for 13–17 months).
(E) Baseline across SG frequency band in CA3 does not change over aging in apoE3-KI mice ($t(18) = 0.18$, $p = 1.0$, n = 12 for 5–8 months, n = 9 for 13–17 months) or apoE4-KI mice (Mann Whitney test with Sidak adjustment, $U = 88$, $p = 0.70$, n = 16 for 5–8 months, n = 14 for 13–17 months).
(F) SD across SG frequency band in CA3 does not change over aging in apoE3-KI mice ($t(18) = 0.50$, $p = 0.95$, n = 12 for 5–8 months, n = 9 for 13–17 months) or apoE4-KI mice (Mann Whitney U test with Sidak adjustment, $U = 98$, $p = 0.93$, n = 16 for 5–8 months, n = 14 for 13–17 months). In A–F, all comparisons unpaired t tests with Sidak adjustment unless otherwise specified.
(G) ApoE4-KI mice show no impairment on MWM at ages 6–9 months, n = 13 apoE3-KI and n = 13 apoE4-KI mice.
(H) CA3 SG power during SWRs measured at 5–8 months predicts slope of escape latency over hidden days 1–3 ($F(1,9) = 10.89$ on MWM task at 14–18 months, n = 11 apoE4-KI mice, Pearson correlation, Holm-Sidak multiplicity adjusted $p = 0.027$.
(I) CA3 SG power during SWRs measured at 5–8 months predicts SWR abundance in the same mouse at 13–17 months for apoE4-KI ($F(1,13) = 27.19$, n = 15) but not apoE3-KI mice ($F(1,7) = 2.52$, n = 9), Pearson correlations.
*$p < 0.05$; **$p < 0.01$; ***$p < 0.001$; ****$p < 0.0001$. Error bars indicate mean ± SEM. Results drawn from 1 independent experiment.

Table S1. Sample sizes for all experiments. Related to STAR Methods.

Age	Metric	Cohort 1		Cohort 2	
		apoE3-KI	apoE4-KI	apoE3-KI	apoE4-KI
5–8 months	SWR Abundance	--	--	13	17[a]
	CA3 SG Power	--	--	12	16
	MWM	--	--	13	13
9–11 months	SWR Abundance	--	--	12[b]	17
	CA3 SG Power	--	--	11[b]	16
12–20 months	SWR Abundance	13	16	9[c]	15[d]
	CA3 SG Power	11	13	9[c]	14[d]
	MWM, Open Field, Elevated Plus	20	19	12[e]	15[f]
	APA, Hot Plate	--	--	12[e]	13[g]

[a]4 apoE4-KI mice aged 7 months were added to the study after the first MWM had been completed.
[b]Implant no longer functional in 1 apoE3-KI mouse
[c]Implant no longer functional in 2 apoE3-KI mice, 1 apoE3-KI mouse died
[d]Implant no longer functional in 1 apoE4-KI mouse, 1 apoE4-KI mouse died
[e]3 apoE3-KI mice died, 3 apoE3-KI mice added
[f]4 apoE4-KI mice died, 3 apoE4-KI mice added
[g]1 apoE4-KI mouse (added just for behavior) excluded due to motor deficits, 1 apoE4-KI mouse died

Table S3. All APA metric correlations tested in replication cohort. Related to Figure 3.

		SWR Abundance				CA3 SG Power			
Metric	Trial	R	F	DFn, DFd	P value	R	F	DFn, DFd	P value
# of Entrances	1	-0.27	0.64	1, 8	0.45	-0.24	0.43	1, 7	0.53
	2	-0.09	0.07	1, 8	0.80	**-0.91**	**32.5**	**1, 7**	**0.0007**
	3	-0.14	0.16	1, 8	0.70	**-0.76**	**9.49**	**1, 7**	**0.02**
	4	0.19	0.29	1, 8	0.61	**-0.72**	**7.48**	**1, 7**	**0.03**
Latency to 1st Entrance	1	-0.43	1.78	1, 8	0.22	0.01	0	1, 7	0.98
	2	**0.65**	**5.81**	**1, 8**	**0.043**	0.27	0.53	1, 7	0.49
	3	0.16	0.22	1, 8	0.65	0.10	0.08	1, 7	0.79
	4	0.36	1.19	1, 8	0.31	0.37	1.12	1, 7	0.33
Path Length	1	0.14	0.15	1, 8	0.71	0.00	0	1, 7	1
	2	**0.82**	**16.42**	**1, 8**	**0.004**	-0.03	0.01	1, 7	0.93
	3	0.23	0.44	1, 8	0.52	-0.28	0.61	1, 7	0.46
	4	0.53	3.08	1, 8	0.12	-0.25	0.45	1, 7	0.53
% Time in Opposite Quadrant	1	0.22	0.39	1, 8	0.55	0.45	1.77	1, 7	0.22
	2	**0.69**	**7.33**	**1, 8**	**0.027**	0.22	0.37	1, 7	0.56
	3	-0.10	0.07	1, 8	0.79	0.13	0.12	1, 7	0.74
	4	-0.03	0.01	1, 8	0.93	0.07	0.04	1, 7	0.85
Dist from Shock Zone per Bout	1	-0.30	0.77	1, 8	0.41	-0.17	0.21	1, 7	0.66
	2	-0.08	0.05	1, 8	0.83	**-0.75**	**9.04**	**1, 7**	**0.02**
	3	0.05	0.02	1, 8	0.90	**-0.7**	**6.74**	**1, 7**	**0.04**
	4	0.38	1.31	1, 8	0.29	-0.15	0.15	1, 7	0.71

Bold values indicate significant correlations.

Table S4. All behavioral correlations tested with 5–8 month apoE4-KI mouse electrophysiological data. Related to Figure 4.

		SWR Abundance				CA3 SG Power			
Age and Behavior	Metric	R	F	DFn, DFd	P value	R	F	DFn, DFd	P value
6-9 mo MWM	Slope of Escape Latency Days 1-2	0.05	0.02	1,11	0.88	0.06	0.02	1,10	0.85
	Slope of Escape Latency Days 1-3	0.17	0.33	1, 11	0.57	-0.04	0.02	1, 10	0.90
	Escape Latency Day 3	-0.03	0.01	1, 11	0.93	0.04	0.02	1, 10	0.90
	Night 1 Learning	0.22	0.55	1, 11	0.47	-0.01	0.001	1, 10	0.97
	Probe 1 % in Target Quadrant	-0.23	0.61	1, 11	0.45	-0.05	0.02	1, 10	0.88
	Probe 2 % in Target Quadrant	-0.06	0.04	1, 11	0.84	0.09	0.08	1, 10	0.79
	Probe 1 Target Crossings	-0.48	3.36	1, 11	0.09	-0.23	0.56	1, 10	0.47
	Probe 1 Dist to Platform Curve	0.27	0.88	1, 11	0.37	0.14	0.19	1, 10	0.68
	Probe 2 Dist to Platform Curve	-0.31	1.18	1, 11	0.30	-0.21	0.44	1, 10	0.52
14-18 mo MWM	Slope of Escape Latency Days 1-2	0.20	0.42	1,10	0.51	**-0.63**	**5.82**	**1,9**	**0.039**
	Slope of Escape Latency Days 1-3	-0.11	0.12	1, 10	0.74	**-0.74**	**10.89**	**1, 9**	**0.009**
	Escape Latency Day 3	0.19	0.37	1, 10	0.56	**-0.74**	**11.08**	**1, 9**	**0.009**
	Night 1 Learning	-0.16	0.27	1, 10	0.62	-0.45	2.30	1, 9	0.16
	Probe 1 % in Target Quadrant	-0.49	3.20	1, 10	0.10	-0.35	1.28	1, 9	0.29
	Probe 2 % in Target Quadrant	-0.31	1.08	1, 10	0.32	-0.07	0.05	1, 9	0.83
	Probe 1 Target Crossings	-0.43	2.21	1, 10	0.17	-0.28	0.77	1, 9	0.40
	Probe 1 Dist to Platform Curve	0.45	2.53	1, 10	0.14	0.22	0.45	1, 9	0.52
	Probe 2 Dist to Platform Curve	0.17	0.29	1, 10	0.60	0.37	1.40	1, 9	0.27
15-20 mo APA Trial 2	# of Entrances	0.60	5.09	1, 9	0.05	-0.09	0.07	1, 8	0.80
	Latency to 1st Entrance	-0.29	0.81	1, 9	0.39	**0.79**	**13.21**	**1, 8**	**0.007**
	Path Length	0.01	0.0003	1, 9	0.99	**0.68**	**6.74**	**1, 8**	**0.03**
	% Time in Opposite Quadrant	-0.18	0.31	1, 9	0.59	0.59	4.25	1, 8	0.07
	Dist from Shock Zone per Bout	0.46	2.48	1,9	0.15	0.11	0.09	1,8	0.77
Trial 3	Dist from Shock Zone per Bout	0.43	2.02	1,9	0.19	0.20	0.33	1,8	0.58

Bold values indicate significant correlations.
